# The Effect of a Horizontal Apical Mattress Suturing Technique on the Width and Thickness of the Keratinized Mucosa After Dental Implant Surgery: A Cohort Study

**DOI:** 10.7759/cureus.65513

**Published:** 2024-07-27

**Authors:** Tarek Khaled Abou-Agwa, Azzam Al-Jundi, Yasser Khaldoun Alhalaby, Hamzah Majed Alakari, Mohammad Y. Hajeer

**Affiliations:** 1 Department of Oral Surgery, College of Dentistry, Al-Wataniya Private University, Hama, SYR; 2 Department of Orthodontics, Faculty of Dentistry, University of Hama, Hama, SYR; 3 Department of Orthodontics, Faculty of Dentistry, University of Damascus, Damascus, SYR

**Keywords:** choukroun's technique, soft-tissue surgery around implants, teeth missing, dental implant surgery, thickness of the keratinized gingiva, width of the keratinized gingiva, keratinized gingiva, keratinized mucosa, horizontal apical mattress suturing technique

## Abstract

Introduction

There is general agreement that a thick zone of the keratinized tissues around implants promotes accurate prosthetic procedures, permits oral hygiene maintenance, resists recession, and enables esthetic blending with surrounding tissues. A new procedure called Choukroun's technique has been suggested, and it consists of a combination of horizontal apical mattress suture with regular suture to increase the keratinized tissue in the mandibular arch during the first stage after implantation. The proposed procedure has not been evaluated yet in a cohort of patients. Therefore, this prospective study aimed to evaluate the impact of Choukroun's technique on the width and thickness of the keratinized gingiva after oral surgery.

Materials and methods

A one-group prospective cohort study was conducted on patients referred to the Department of Oral and Maxillofacial Surgery at the Dental College of Al-Wataniya Private University who had been referred to undergo dental implant surgery. The inclusion criteria for the study group were as follows: patients with mandibular missing teeth, good oral health, good general health, 18-70 years old, at least 1 mm thickness of keratinized gingiva, and at least 1 mm width of the attached gingiva. A total of 14 patients aged 27-67 years were included in the study. After inserting the dental implants, the suturing was accomplished according to Choukroun's method. The width and thickness of the keratinized gingiva were assessed before surgery and at one and two months post-surgery. Repeated measures analysis of variance (ANOVA) was applied to detect significant differences between assessment times.

Results

The study sample comprised 14 patients, of which four were females (28.6%) and 10 were males (71.4%). Patients' ages ranged from 27 to 67 years, with a mean age of 54.86 ± 11.73 years. The surgical procedure was performed in three different regions: the upper posterior teeth with four patients (28.6%), the upper anterior teeth with three patients (21.4%), and the lower posterior teeth with seven patients (50%). The mean gingival width before surgery was 5.78 mm, whereas the mean gingival thickness was 2.82 mm. There was no significant difference between the three evaluation times in the mean gingival width (P=0.222), and there was a significant difference between the three evaluation times in the mean gingival thickness (P<0.001). The mean mean gingival thickness one month after surgery was significantly greater than its mean value before surgery (mean difference: 0.749 mm). Additionally, the mean gingival thickness two months after surgery was significantly greater than its mean value before (mean differences: 0.636 mm).

Conclusions

Using horizontal apical mattress sutures (Choukroun's technique) does not provide any advantage in increasing the width of the keratinized gingiva. However, it does lead to an increase in the thickness of keratinized gingiva in the surgical area. Therefore, the use of horizontal apical mattress sutures is recommended.

## Introduction

When employed correctly, surgical sutures are instrumental in maintaining the alignment of flap edges until the wound has sufficiently healed to endure normal functional stresses [1]. Applying the proper suture technique, combined with the appropriate thread type and diameter, places tension on the wound margins, facilitating primary intention healing [1]. However, when surgical wound edges are inadequately aligned, insufficient apposition can result in hemostasis, allowing for the accumulation of blood and serum beneath the flap. This, in turn, delays the healing process by causing a separation between the flap and the underlying bone [2].

Suture materials play an important role in wound healing, enabling the reconstruction and reassembly of tissue separated by a surgical procedure or trauma and facilitating and promoting healing and hemostasis [3]. The development of synthetic suture materials introduced a few suture materials of different characteristics, good quality, and acceptable price [4]. However, the ideal suture material has not been manufactured yet.

Crucial features of absorbable suture materials include their absorption process and the gradual loss of tensile strength over time. These factors are critical in determining whether the absorbable suture material will remain intact and provide sufficient strength to support and promote wound healing [5]. Superficial wound tissue typically takes five to 10 days to heal, while certain surgical procedures necessitate sutures that remain in place for 14-28 days [6]. Nevertheless, given that absorbable suture materials dissolve over time due to proteolytic enzymes or hydrolysis, it is preferable not to leave them in tissue longer than necessary [5]. The selection of the most suitable suture material depends on the location and depth of the tissue to be stitched [7].

Comparatively, the monofilament synthetic absorbable suture exhibits significantly greater strength than its braided counterpart over a four-week implantation period. Additionally, the monofilament suture is associated with lower bacterial infection rates than the braided suture [8]. A mattress means the suture passes through the flap twice. Horizontal mattress sutures are used when more precise apposition of wound edges is needed. Horizontal sutures have less tendency to tear through tissue [9].

However, it is worth noting that the horizontal mattress suture is a secondary suture line placed away from the wound edges. Therefore, additional sutures are needed to unite the wound edges, allowing primary intention healing. This type of suture can be left in place for an extended period, as in augmentation procedures [10]. Placing the near insertion point close to the wound margin enhances the precision of wound margin approximation [11]. After the periosteum is elevated, it takes a considerable amount of time for it to reattach to the bone, with 30 days needed to achieve 70% reattachment and 45 days for significant contact between the periosteum and bone to take place [12]. The minimum amount of keratinized tissue needed to sustain periodontal health is disputed, and there may be no minimum in the absence of plaque and inflammation [13].

Bouri et al. [14] reported that the absence of an adequate amount of keratinized mucosa around dental implants, especially in the posterior region, was associated with higher plaque accumulation and gingival inflammation. This was in agreement with a cross-sectional study done by Akolu et al. [15], who concluded that increased width of keratinized mucosa (>2 mm) around implants is associated with lower mean alveolar bone loss and improved indices of soft tissue health.

There is general agreement that a thick zone of the keratinized tissues around implants promotes accurate prosthetic procedures, permits oral hygiene maintenance, resists recession, and enables esthetic blending with surrounding tissues. Soft tissue augmentation around implants can be performed at various stages of implant therapy [16]. A new procedure called Choukroun's technique has been suggested, and it consists of a combination of horizontal apical mattress sutures with regular sutures to increase the keratinized tissue in the mandibular arch during the first stage of implant surgery [17]. The proposed procedure has not been evaluated yet in a group of patients. Therefore, this cohort study aimed to evaluate the impact of Choukroun's technique on the width and thickness of the keratinized gingiva after oral surgery.

## Materials and methods

Study design and settings

A cohort study was accomplished to assess the effect of a modified suturing technique on the width and thickness of keratinized gingiva following dental implant placement surgery. Patients were monitored during three assessment times: pre-surgery, one-month post surgery, and two months post surgery. The study protocol was approved by the Local Research Ethics Committee of Al-Wataniya Private University (ID: 88/b/8/2022) and funded by Al-Wataniya Private University (Ref No.: 32154DEN/2022).

Patient recruitment

The study included 14 patients aged 27-67. All the patients referred to undergo dental implant surgery were recruited from the Department of Oral and Maxillofacial Surgery attendees at the Dental College of Al-Wataniya Private University. The study started in September 2022 and finished in April 2023.

The inclusion criteria for the study group were as follows: patients with mandibular missing teeth, good oral health, good general health, male or female, age between 18 and 70 years old, thickness of keratinized gingiva at least 1 mm, and width of attached gingiva at least 1 mm. The study group's exclusion criteria were as follows: patients should be nonsmokers, not pregnant or lactating, have no systemic diseases, have not taken medication in the last six months that could influence wound healing after surgery, and have no oral inflammation, such as periodontal disease.

**Figure 1:**
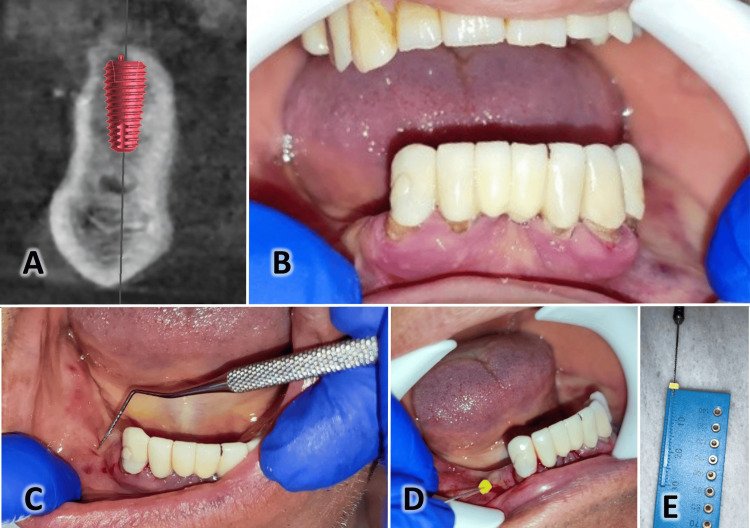
Pre-surgical evaluation of a case from this cohort of patients. A: A sagittal view of the CBCT image illustrating the bone condition with the virtual dental implant. B: An intraoral image before surgery. C: Measuring the width of the attached gingiva using a periodontal probe. D: Measuring the thickness of the keratinized gingiva by inserting an endodontic file with a rubber tip. E: Converting the measured distance on the endodontic file into a number using a ruler.

Before the surgical procedure, each patient received information about the surgery, postoperative recommendations, and possible complications. They signed a consent form indicating their agreement to participate in the study. Figure 1 presents a case requiring multiple implants in the lower jaw with a preliminary assessment. This included the measurement of the width and thickness of the keratinized gingiva.

Surgical procedure and suturing technique

Patients were pre-medicated with amoxicillin + clavulanic acid (2 g) one hour before surgery. Following surgery, 875 mg of amoxicillin + 125 mg of clavulanic acid was administered twice a day for one week. The oral cavity was rinsed with a 0.12% chlorhexidine gluconate solution (Biofresh®, Melqart Pharma, Damascus, Syria) for one minute, and the skin surrounding the mouth site was disinfected. All operations were performed under local anesthesia by the same oral surgeon (TKA, the principal researcher), using 2% lidocaine with 1:80,000 epinephrine.

Surgical intervention

The flap was designed to provide a clear view of the surgical area and to ensure primary tension-free closure. A mid-crestal incision was made in the keratinized gingiva on the alveolar crest, and a sulcular incision was used to release the gingiva around the remaining teeth if needed. Full-thickness mucoperiosteal flaps were released. After routine implant placement, the cover screw has been placed, and then the following suturing technique was performed according to Choukroun's technique [17] using Vicryl suture 5-0; this technique is described elsewhere. Implants were placed in suitable positions without using bone grafts and membranes.

**Figure 2:**
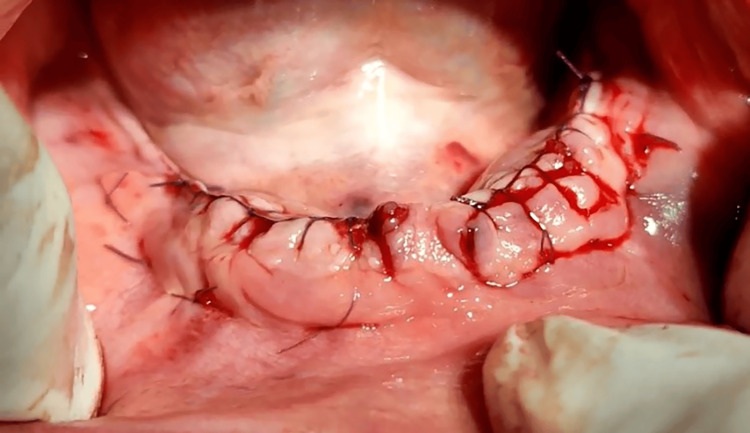
An intra-oral image of the lower jaw following the dental implant surgery and the performance of the suturing technique.

Choukroun's technique involves several steps. Firstly, the buccal flap is penetrated apically at a 1 to 1.5 cm depth from the margin. Then, it passes through the lingual flap from the inside surface and returns from the outside surface of the lingual flap. Additionally, passing through the inside surface of the buccal flap at the same level as the first point. It is worth noting that the distance between point 1 and point 4 and between point 2 and point 3 is 3 mm. A traditional surgical knot is used. This step aids in achieving tension-free closure of deeper tissues while providing wound edge eversion. After completing the apical mattress sutures, individual interrupted sutures are performed at the margin of the incision to ensure precise wound closure (Figure 2). The sutures are left in place for four weeks.

**Figure 3:**
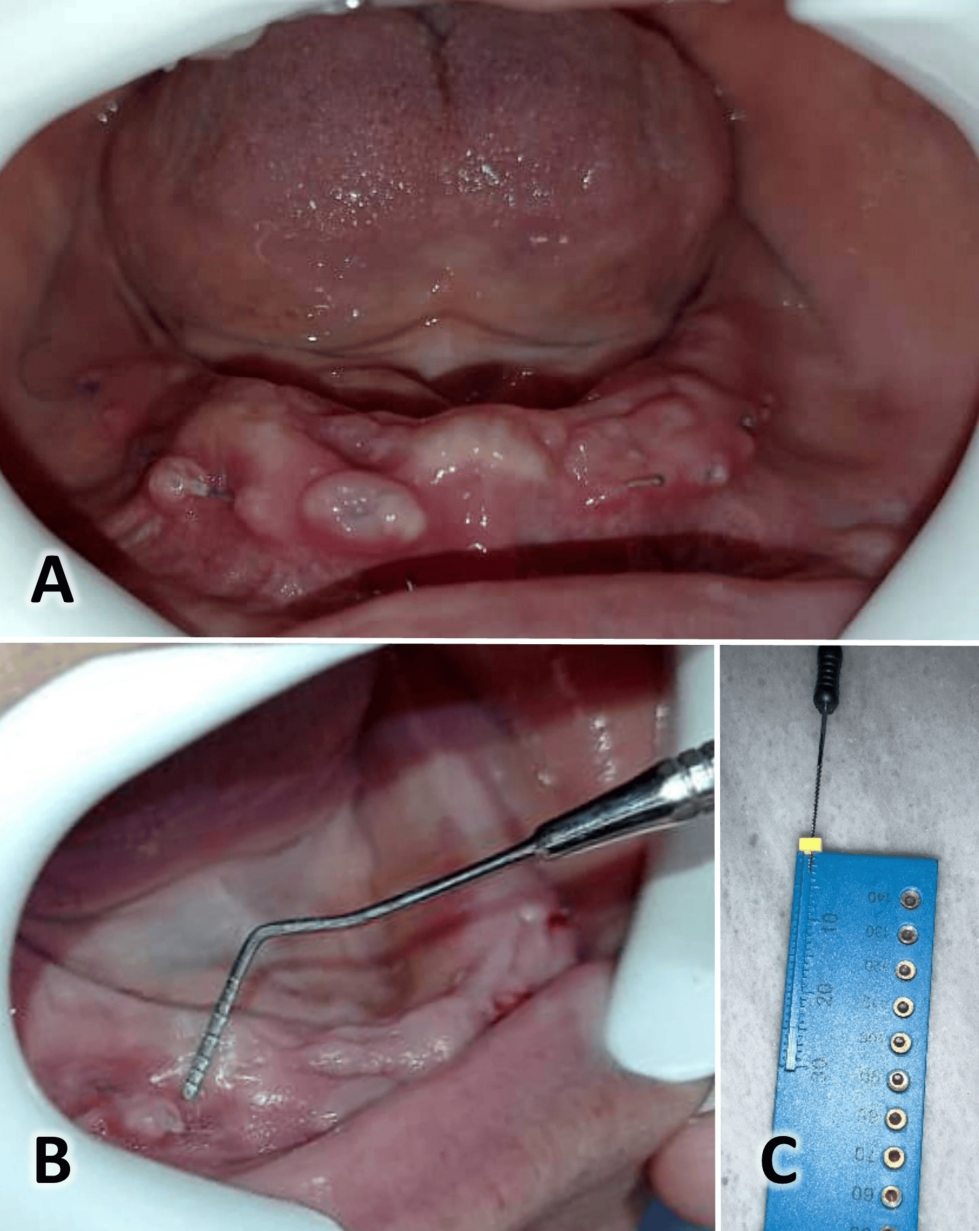
One-month postoperative evaluation of the case. A: An intraoral image of the lower jaw. B: Measuring the width of the keratinized gingiva. C: Measuring the thickness of the keratinized gingiva.

**Figure 4:**
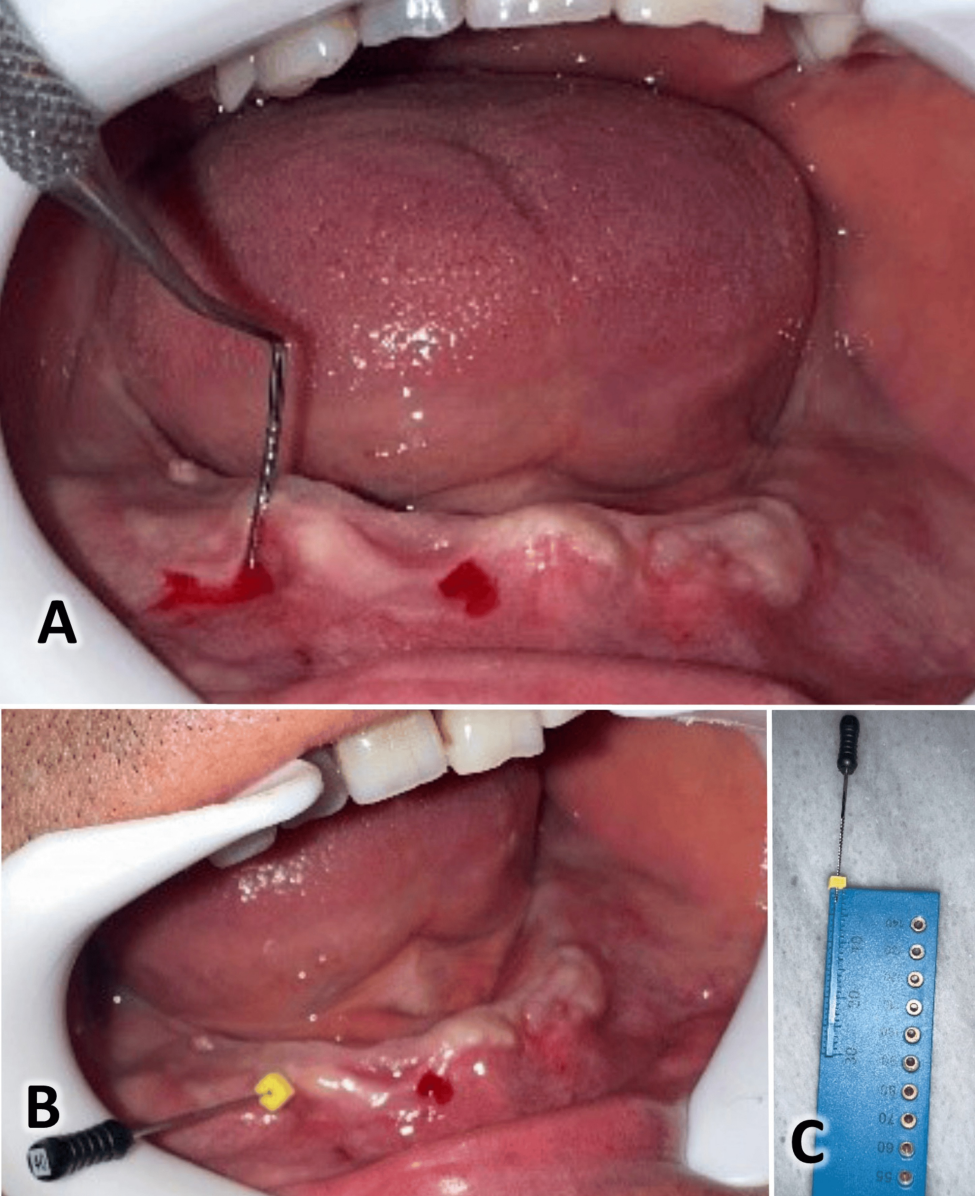
Two-month postoperative evaluation of the case. A: The width of the keratinized gingiva. B: Measuring the width of the keratinized gingiva. C: Measuring the thickness of the keratinized gingiva.

**Figure 5:**
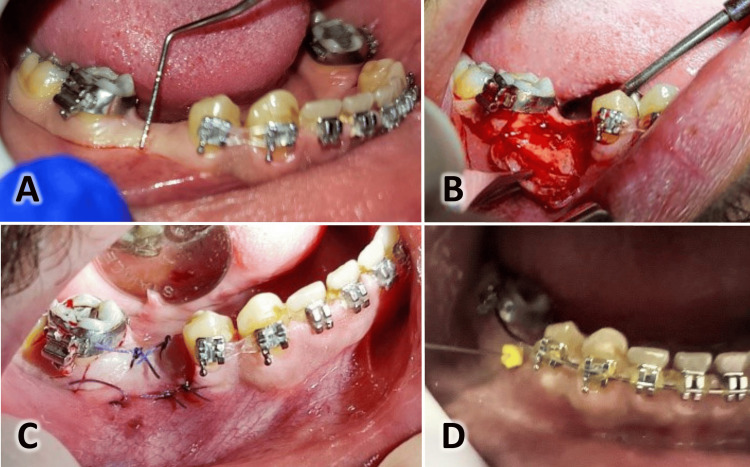
An orthodontic patient who required an implant to replace the missing lower second premolar. A: An intraoral image of the case showing the measurement of the width of the keratinized gingiva before dental implant surgery. B: Flap elevation during surgery. C: The suturing after implant insertion. D: An intraoral image at two months postoperatively showing the measurement of the thickness of the keratinized gingiva.

All patients received postoperative instructions, including using ice packs six hours after surgery, a soft, warm diet for the first 24 hours, normal oral hygiene from the day after surgery, and mouthwash with 0.12% chlorhexidine twice daily for one week. Patients were prescribed antibiotics (875 mg of amoxicillin + 125 mg of clavulanic acid) and analgesic drugs (ibuprofen 200 mg per day for three days). The length of the operation was also noted. Figures 3-4 present the same case shown in Figure 1 at the one-month and two-month assessments. Another case is shown in Figure 5, where the suturing of the raised flaps was accomplished using the same Choukroun technique, with good results achieved after two months.

Outcome measures

Measurements were taken before the surgical procedure (T0) using a periodontal probe (William probe), an endodontic file, and a ruler. The width of the attached gingiva was measured at the mesial, distal, and middle positions within the surgical area (from the crest of the alveolar ridge to the mucogingival junction), and the mean of these measurements was recorded. To measure the thickness of the keratinized gingiva, an endodontic file equipped with a rubber tip was used to penetrate the gingiva at various points within the surgical area until it reached the bone surface. These points are located midway between the mid-crestal of keratinized gingiva and the mucogingival junction, spaced 2 mm apart, and extending from the mesial to the distal aspects of the surgical area. The thickness was then measured using a ruler, and the mean of these values was recorded. All measurements were taken by the same principal researcher (TKA). The width and thickness of keratinized gingiva were measured using the same method one month (T1) and two months (T2) after the surgical procedure.

Statistical analysis

The recorded data were analyzed using the Statistical Product and Service Solutions (SPSS, version 23.0; IBM Corp, Armonk, NY). Repeated-measures analysis of variance (ANOVA) tests were employed, and Bonferroni's post-hoc tests were performed when needed.

## Results

Baseline sample characteristics

This study sample comprised four female patients (28.6%) and 10 male patients (71.4%), aged between 27 and 67 years, with a mean age of 54.86 years and a standard deviation of 11.73 years. All patients underwent oral surgery from September to December 2022. The surgical procedure was performed in three different regions: the upper posterior teeth with four patients (28.6%), the upper anterior teeth with three patients (21.4%), and the lower posterior teeth with seven patients (50%), as shown in Table 1.

**Table 1:** The baseline sample characteristics in terms of gender, age, and location of surgery.

Variable	N (%)/mean (SD)
Gender	
Male	12 (71.4)
Female	4 (28.6)
Age	54.86 ± 11.73
Location of the surgical intervention	
Lower posterior teeth	7 (50.0)
Upper posterior teeth	4 (28.6)
Upper anterior teeth	3 (21.4)

Descriptive statistics of the main variables

Table 2 gives the descriptive statistics of the measured variables for the whole sample (i.e., n=14). The mean gingival width before surgery was 5.78 mm, whereas the mean gingival thickness was 2.82 mm. One month after surgery, the mean gingival width was 5.85 mm, whereas the mean gingival thickness increased to 3.57 mm. The results showed no significant difference between the three evaluation times in the mean gingival width (P=0.222). There was a significant difference between the three evaluation times in the mean gingival thickness (P<0.001).

**Table 2:** Descriptive statistics of the average gingival width and thickness measurements and the significance of changes over time. T0: before surgery, T1: one month after surgery, T2: two months after surgery; SD: Standard deviation ^a^Repeated-measures ANOVA. ^*^Significant at the 0.05 level.

Variable		Mean	Median	SD	Range	Min	Max	F	P-value^a^
Gingival width	T0	5.78	5.15	1.20	4.33	4.67	9.00	0.872	0.222
	T1	5.85	5.50	1.07	3.80	5.00	8.80		
	T2	5.77	5.15	1.17	4.18	4.72	8.90		
Gingival thickness	T0	2.82	2.50	1.00	3.00	1.67	4.67	145.787	<0.001*
	T1	3.57	3.30	0.93	2.72	2.50	5.22		
	T2	3.45	3.20	0.92	2.73	2.37	5.10		

Pairwise comparisons were conducted to detect the time comparison that had a significant difference. The mean gingival thickness one month after surgery was significantly greater than that before surgery, with a mean difference of 0.749 mm. The mean gingival thickness two months after surgery was significantly greater than before, with a mean difference of 0.636 mm (Table 3).

**Table 3:** Pairwise comparisons of the gingival thickness. T0: before surgery, T1: one month after surgery, T2: two months after surgery ^a^Bonferroni's post-hoc test. ^*^Significant at the 0.05 level.

Variable	Time Comparison	Mean Difference	P-value^a^
Gingival thickness	T0 VS T1	-0.749	<0.001*
	T0 VS T2	-0.636	<0.001*
	T1 VS T2	0.113	0.009*

The results showed that age did not affect the variations in gingival thickness during treatment (P=0.402). There was no significant difference between the two genders in gingival thickness during the treatment period (P=0.670). In addition, there was no difference between the surgical regions in gingival thickness during the treatment period (P=0.539).

## Discussion

Precise flap closure is a major determining factor in achieving wound healing by primary intention and obtaining the desired treatment result [18]. Horizontal mattress suturing can improve primary wound healing in impact mandibular third molar surgery [19]. Choukroun has introduced the suturing technique through his website [17]. This technique combines a single interrupted suture and a horizontal mattress suture. Wachtel et al. found that combining single interrupted sutures and tension-relieving sutures (such as mattress sutures) makes it possible to achieve very good and stable flap adaptation with relatively little time and effort [20,21]. The horizontal mattress suture in the Choukroun technique involves a modification in the distance of the buccal insertion points of the needle, ensuring that the buccal points are positioned approximately 1-1.5 cm away from the margin of the incision. In the traditional horizontal mattress, sutures are initiated by inserting the needle slightly farther from the wound edge than by placing simple interrupted sutures.

This study aimed to investigate the influence of the Choukroun horizontal mattress suturing technique (with 5/0 absorbable sutures) on the width and thickness of keratinized mucosa after implant surgery. Accurate apposition of the surgical flaps enables hemostasis, reduces the wound size to be repaired, prevents unnecessary bone destruction, and improves patient comfort [22]. If surgical wound edges are not properly approximated and dead spaces are present, blood may accumulate under the flap, which delays the healing process by separating the flap from the underlying bone [22]. Depending on that, Choukroun's technique (as a horizontal mattress suture) contributed to the accurate apposition of the surgical flaps and enhanced periosteal attachment to the bone by keeping the flap attached to the underlying bone. That was proved by Burkhardt et al., who found that horizontal mattress suture firmly adapts the soft tissue flap to the underlying structures [22].

The absence of adequate keratinized tissue around implants is associated with higher plaque accumulation, gingival inflammation, bleeding on probing, and mucosal recession [23]. Keratinized tissue around implant-supported overdentures affects bone maintenance and soft tissue health around those implants [24].

Various surgical procedures have been developed to preserve and/or reconstruct keratinized tissue around dental implants [23,24]. These techniques, including apically positioned flaps, pedicle grafts, free gingival grafts, and connective tissue grafts, can be performed before implant placement, during the second stage of surgery, or after delivery of the final prosthesis. Allogenic and xenogenic soft tissue grafts have also been used as other options for increasing peri-implant keratinized tissue [25-27].

In Choukroun's technique, the keratinized mucosa was displaced coronally under the influence of the tension forces resulting from the suturing, thereby increasing the thickness of the keratinized gingival tissues. This effect persists into the postoperative stage, as demonstrated by our study. Ultimately, this study found strong correlations in gingival thickness after two months for individuals aged between 30 and 67. The increased thickness was about 0.93 mm one month after surgery and 0.92 mm two months after.

Limitations of the current work

The current study included patients with a very large age range. This cannot help generate the results for other specific age groups. Surgical procedures were limited to the lower jaw, and we recommend applying the technique to the upper jaw. Additionally, the current sample size is small, and larger samples are required for robust results.

## Conclusions

The findings of our study showed that using the horizontal apical mattress suture (Choukroun's technique) does not provide any advantage in increasing the width of the keratinized gingiva. However, it does lead to an increase in the thickness of keratinized gingiva in the surgical area. This increase in gingival thickness can improve tissue stability and potentially enhance the overall outcome of periodontal surgeries. The increase in thickness can also provide better resistance to mechanical trauma and may improve the esthetic appearance of the gingiva. Therefore, horizontal apical mattress sutures are recommended for situations where an increase in gingival thickness is desired despite the lack of effect on gingival width. This technique can be particularly beneficial in cases where enhanced tissue support and durability are critical for the success of the surgical procedure.
